# Falls among Brazilian older adults living in urban areas: ELSI-Brazil

**DOI:** 10.11606/S1518-8787.2018052000635

**Published:** 2018-10-25

**Authors:** Wendel Rodrigo Teixeira Pimentel, Valéria Pagotto, Sheila Rizzato Stopa, Maria Cristina Corrêa Lopes Hoffmann, Fabíola Bof de Andrade, Paulo Roberto Borges de Souza, Maria Fernanda Lima-Costa, Ruth Losada de Menezes

**Affiliations:** I Universidade de Brasília. Faculdade de Ceilândia. Programa de Pós-graduação em Ciências e Tecnologias em Saúde. Brasília, DF, Brasil; II Universidade Federal de Goiás. Faculdade de Enfermagem. Goiânia, GO, Brasil; III Universidade de São Paulo. Faculdade de Saúde Pública. Programa de Pós-Graduação em Saúde Pública. São Paulo, SP, Brasil; IV Ministério da Saúde. Secretaria de Atenção à Saúde. Coordenação de Saúde da Pessoa Idosa. Brasília, DF, Brasil; V Fundação Oswaldo Cruz. Instituto René Rachou. Núcleo de Estudos em Saúde Pública e Envelhecimento. Belo Horizonte, MG, Brasil; VI Fundação Oswaldo Cruz. Instituto René Rachou. Programa de Pós-Graduação em Saúde Coletiva. Belo Horizonte, MG, Brasil; VII Fundação Oswaldo Cruz. Instituto de Comunicação e Informação Científica e Tecnológica em Saúde. Rio de Janeiro, RJ, Brasil

**Keywords:** Aged, Accidental Falls, Socioeconomic Factors, Epidemiological Surveys, Associated Factors, Idoso, Acidentes por Quedas, Fatores Socioeconômicos, Inquéritos Epidemiológicos, Fatores Associados

## Abstract

**OBJECTIVE:**

To assess the prevalence and factors associated with falls in a nationally representative sample of older Brazilians residing in urban areas.

**METHODS:**

Data from 4,174 participants (60 years or older) from the baseline of ELSI-Brazil, conducted between 2015 and 2016, were used. The outcome variable was the reporting of one or more falls in the last 12 months. The exploratory variables were sociodemographic characteristics, factors related to the urban environment, and health conditions. Statistical analysis was performed using Poisson regression.

**RESULTS:**

The prevalence of falls was 25.1%. Of these, 1.8% resulted in a hip or femur fracture and, among them, 31.8% required surgery for prosthesis placement. Statistically significant associations (p < 0.05) with falls were observed for females [prevalence ratio (PR) = 1.26], age group of 75 years or older (PR = 1.21), fear of falling due to defective sidewalks (PR = 1.47), fear of crossing streets (PR = 1.22), diabetes (PR = 1.17), arthritis or rheumatism (PR = 1.29), and depression (PR = 1.53). No significant associations were found for educational level, marital status, hypertension, and perception of violence in the neighborhood.

**CONCLUSIONS:**

The factors associated with falls among older adults are multidimensional, comprising individual characteristics and the urban environment, which indicates the need for intra and intersectoral actions to prevent falls in this population.

## RESUMO

**OBJETIVO:**

Determinar a prevalência e os fatores associados a quedas em amostra nacional representativa da população idosa residente em áreas urbanas.

**MÉTODOS:**

Foram utilizados dados de 4.174 participantes (60 anos ou mais) da linha de base do ELSI-Brasil, conduzida entre 2015 e 2016. A variável de desfecho foi o relato de uma ou mais quedas nos últimos 12 meses. As variáveis exploratórias foram características sociodemográficas, fatores relacionados ao ambiente urbano e condições de saúde. A análise estatística foi realizada por meio da regressão de Poisson.

**RESULTADOS:**

A prevalência de quedas foi de 25,1%. Destas, 1,8% resultaram em fratura de quadril ou fêmur e, entre elas, 31,8% necessitaram de cirurgia com colocação de prótese. Após ajustes pertinentes, associações estatisticamente significantes (p < 0,05) com a ocorrência de quedas foram observadas para o sexo feminino [razão de prevalência (RP) = 1,26], faixa etária igual ou superior a 75 anos (RP = 1,21), medo de cair devido a defeitos nos passeios (RP = 1,47), medo de atravessar a rua (RP = 1,22), diabetes (RP = 1,17), artrite ou reumatismo (RP = 1,29) e depressão (RP = 1,53). Não foram observadas associações significativas para o nível de escolaridade, a situação conjugal, a hipertensão e a percepção da violência na região de vizinhança.

**CONCLUSÕES:**

Os fatores associados às quedas entre idosos são multidimensionais, incluindo características individuais e o ambiente urbano, o que indica a necessidade de ações intra e intersetoriais para a prevenção de quedas nessa população.

## INTRODUCTION

Falls are considered a public health problem, given their prevalence and repercussions for the health of the world’s aged population. People of all ages are at risk of falling. However, for older adults, a fall represents a particularly relevant event, since this event may restrict activities of daily living, cause fear of falling again, fractures and hospitalizations, leading to an increased risk of disability and mortality^1^.

The proportion of older people falling differs between countries. In the Americas, the prevalence of falls in community-dwelling older adults varies between 20% in Canada^2^ and 35% in Chile^3^. Among the Europeans, 28.4% of the English^4^ reported having fallen in the last two years and 19.4% of Irish^5^ had fallen in the last year. In Brazil, the prevalence of falls in the previous year varies between 10% and 35%, according to the population studied^6^ ^-^ ^10^. Different risk factors for the occurrence of falls in older adults may act alone or in association with other factors. Studies associations of falls with female gender^2^ ^,^ ^4^ ^,^ ^6^ ^,^ ^7^, advanced age^4^ ^-^ ^6^ ^,^ ^10^, marital status (divorced), living alone^2^ ^,^ ^11^, fear of falls^12^ ^,^ ^13^, poor rated health ^10^ ^,^ ^14^, depression^15^, use of medication on regular basis^10^, chronic diseases^4^, worse vision or hearing^16^ ^,^ ^17^, among other health problems, and inadequate environmental factors^8^.

The prevention of this problem represents a great challenge for older adults, the family, the community, professionals, and health systems^1^, among them the public health system in Brazil (Unified Health System -SUS). Information on this subject is essential to subsidize decisions about prevention and actions, as well as to plan public policies for this population. Although there were several studies on falls^6^ ^-^ ^11^, only one of them^9^ was conducted in a representative sample of the Brazilian older population. This study was conducted in 2009 and showed a prevalence of falls that varied between 18% in the North region and 20% in the Southeast region. A recent review of the literature points to the need to carry out new Brazilian studies on the subject^18^.

The present study aimed to assess the prevalence of falls among Brazilian older adults living in urban areas and to examine their association with sociodemographic characteristics, factors related to the urban environment, and health conditions.

## METHODS

Data from the baseline of the Brazilian Longitudinal Study of Aging (ELSI-Brazil), conducted between 2015 and 2016, were used. The sample was designed to represent the non-institutionalized Brazilian population aged 50 years or older. It is a complex sample, based on different stages of selection, including municipality, census tract, and household. The research was conducted in 70 municipalities located in different regions of the country. The baseline of the study was made up of all residents in the sampled households, in the mentioned age group. The sample size was estimated at 10,000 individuals (9,412 participated). Among the participants, 85% lived in urban areas, according to the classification of the Brazilian Institute of Geography and Statistics (IBGE), similar to that observed for the Brazilian population at the corresponding age^19^. For the present analysis, all the participants of the survey, aged 60 years and older, living in an urban area (n = 4,533) were eligible. More details on the ELSI-Brazil can be found on the research homepage^a^ and in a previous publication^19^.

The dependent variable of the study was the occurrence of one or more falls, the information of which was obtained from the question: “In the last 12 months, did you have any falls?” Fall was defined as “an unintentional displacement of the body to a level lower than the initial position, incapable of correction in a timely manner, as determined by multifactorial conditions that compromise stability”^20^. Among the descriptive variables, we also considered hip or femur fractures and the need for surgery for prosthesis placement.

The independent variables included sociodemographic characteristics, factors related to the urban environment, and health conditions. The sociodemographic characteristics considered were: gender; age group (60-64, 65-74, and 75 years or older); marital status (married, single or divorced or separated, and widower); (illiterate, 1-4, 5-8, and nine years or more of formal schooling). Among the factors of the urban environment, we considered: fear of falling due defective sidewalks; afraid to cross the street; and perception of neighborhood security in relation to violence. The indicators of health condition considered were previous medical diagnosis of hypertension, diabetes, arthritis or rheumatism, and depression.

Statistical analyzes of the associations between the independent variables and the occurrence of falls were based on estimates of prevalence ratios estimated by using Poisson regression. Initially, the associations of each independent variable with the outcome were examined by adjustments for age and gender. Subsequently, the final multivariate analysis was performed, with mutual adjustments for all the covariates already described. These variables entered simultaneously into the final multivariate model since they did not show evidence of collinearity (Variance inflation factor < 5.0). The analyzes were performed using the statistical package Stata, version 14.0, using the survey module (svy), which considers the effects of the complex sample.

ELSI-Brazil was approved by the Research Ethics Committee of the Fundação Oswaldo Cruz, Minas Gerais (CAAE 34649814.3.0000.5091). All participants signed a free and informed consent form.

## RESULTS

Of the 4,533 participants eligible for the study, 4,174 had complete information for all variables and were included in the analysis. Among these, the average age was 70.2 years (95%CI 69.6–70.7), and female gender predominated (56.6%). Other characteristics of the study participants can be seen in Table 1.

**Table 1:** Characteristic of the 4,174 participants in the sample. Brazilian Longitudinal Study of Aging (ELSI-Brazil), 2015-2016.

Characteristic	%*	95%CI
One or more falls in the last 12 months	25.1	23.6–26.7
Female gender	56.6	53.6–59.5
Age group (years)		
60–64	35.5	33.1–38.1
65–74	38.3	36.4–40.2
75 or older	26.2	23.8–28.7
Marital status		
Married/Common law	15.2	12.4–18.5
Single/Separated	19.0	17.2–21.0
Widowed	23.3	21.2–25.7
Education (years)		
Never studied	15.2	12.4–18.5
1–4	42.3	39.3–45.3
5–8	18.8	17.3–20.4
9 or more	23.8	21.0–26.8
Is afraid of falling due to defective sidewalks	56.1	53.8–58.5
Is afraid to cross streets	48.9	46.2–51.6
Perceives the neighborhood as very insecure	35.5	32.3–38.9
Hypertension diagnosed by a physician	61.3	58.8–63.8
Diabetes diagnosed by a physician	20.2	18.7–21.8
Arthritis or rheumatism diagnosed by a physician	25.0	23.2–26.9
Depression diagnosed by a physician	17.6	15.7–19.6

* Weighted by individuals’ weights and sample parameters.

The occurrence of at least one fall in the last 12 months was reported by 25.1% (95%CI 23.6–26.7) of the participants. This prevalence was higher among women (30.2%, 95%CI 28.3–32.2) than men (18.4%, 95%CI 16.2–20.8). At the time of these falls, 1.8% (95%CI 1.1–2.9) fractured the hip or the femur, of which 31.8% (95%CI 13.4–58.4) required surgery for prosthesis placement. For both genders, the occurrence of falls was greater in the age group of 75 years or older (Figure).

**Figure:**
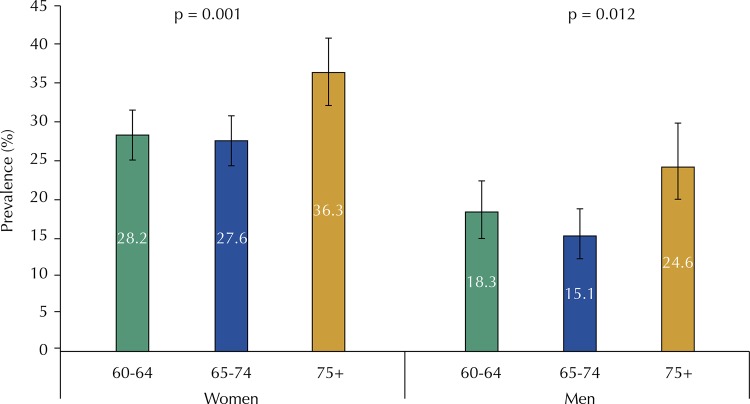
Prevalence of one or more falls in the last 12 months, according to gender and age group. Brazilian Longitudinal Study of Aging (ELSI-Brazil), 2015-2016.

^a^ Pearson’s chi-squared test

^b^ P-value for differences between age groups (Pearson’s chi-squared test)

Table 2 shows the results of the age-gender adjusted analysis of the association between falls and sociodemographic characteristics. Statistically significant associations were observed only for female gender and age group 75 years and older.

**Table 2:** Prevalence of one or more falls in the last 12 months and its association with sociodemographic characteristics. Brazilian Longitudinal Study of Aging (ELSI-Brazil), 2015-2016.

Characteristic	Prevalence^a^	Prevalence ratio^b^	95%CI
Gender			
Male	18.4	1	
Female	30.2	1.62^c^	1.42–2.85
Age group (years)			
60–64	23.6	1	
65–74	22.1	0.93^c^	0.80–1.07
75 or older	31.4	1.29^c^	1.09–1.52
Marital status			
Married/Common law	22.0	1	
Single/Separated	27.0	11.10	0.93–1.33
Widowed	31.1	1.11	0.95–1.29
Education (years)			
Illiterate	27.4	1	
1–4	24.8	0.96	0.83–1.07
5–8	26.7	1.10	0.93–1.32
9 or more	22.8	0.94	0.78–1.14

^a^ Estimates in relation to the total of the column and weighted by the weights of the individuals and sample parameters.

^b^ Adjusted by gender and age group and estimated by Poisson regression.

^c^ Adjusted by age group and vice versa.

As shown in Table 3, in the results of the age-gender adjusted analyzes showed statistically significant associations with the occurrence of falls for: fear of falling due to defective sidewalks, fear of crossing streets, and self-reported diabetes, arthritis or rheumatism, and depression.

**Table 3:** Prevalence of one or more falls in the last 12 months and its association with characteristics of the urban environment and health conditions. Brazilian Longitudinal Study of Aging (ELSI-Brazil), 2015-2016.

Characteristics	Prevalence^a^	Prevalence ratio^b^	95%CI
Is afraid of falling due to defective sidewalks			
No	17.1	1	
Yes	31.4	1.66	1.41–1.95
Is afraid to cross the street			
No	19.3	1	
Yes	31.1	1.46	1.26–1.69
Perceives the neighborhood as very insecure			
No	24.3	1	
Yes	26.6	1.11	0.99–1.24
Hypertension diagnosed by a physician			
No	22.2	1	
Yes	26.9	1.15	1.00–1.32
Diabetes diagnosed by a physician			
No	24.0	1	
Yes	29.5	1.21	1.07–1.38
Arthritis or rheumatism diagnosed by a physician			
No	22.1	1	
Yes	34.2	1.39	1.23–1.57
Depression diagnosed by a physician			
No	21.9	1	
Yes	40.0	1.69	1.47–1.95

^a^ Estimates in relation to the total of the column and weighted by the weights of the individuals and sample parameters.

^b^ Adjusted by gender and age group and estimated by Poisson regression.

The final results of the multivariate analysis are shown in Table 4. In the final model, we observed that falls remained significantly associated with female gender (PR = 1.26), age group 75 years or older (PR = 1.21), fear of falling due to defective sidewalks (PR = 1.47), fear of crossing streets (PR = 1.22), diabetes (PR = 1.17), arthritis or rheumatism (PR = 1.20), and depression (PR = 1.53).

**Table 4:** Statistically significant results of the multivariate analysis of associations between one or more falls in the last 12 months and sociodemographic characteristics, urban environment, and health conditions. Brazilian Longitudinal Study of Aging (ELSI-Brazil), 2015-2016.

Characteristic	Prevalence ratio*	95% CI
Female gender (vs. male)	1.26	1.08–1.48
Age group in years (vs. 60-64)		
65–74	0.94	0.82–1.08
75 or older	1.21	1.01–1.45
Is afraid of falling due to defects on the ground (versus no)	1.47	1.26–1.73
Is afraid to cross the street (versus no)	1.22	1.07–1.40
Diabetes diagnosed by a physician (versus no)	1.17	1.03–1.32
Arthritis or rheumatism diagnosed by a physician (versus no)	1.20	1.06–1.36
Depression diagnosed by a physician (versus no)	1.53	1.32–1.77

* Simultaneously adjusted by all the variables considered in the study, through Poisson regression.

## DISCUSSION

The results of the present study show that, among older people living in urban areas, the factors associated with the occurrence of falls are multidimensional. Regarding sociodemographic characteristics, gender and age showed independent associations with the outcome. However, even in view of the great social disparities observed in the health conditions of the Brazilian older adults^21^, the propensity for falls did not vary by level of education. The urban environment was associated with the occurrence of falls, and those who are afraid to fall due to defective sidewalks and who fear crossing streets were more likely to the outcome. In contrast, the perception of neighborhood security was not associated with the outcome. Among health conditions, diabetes, arthritis or rheumatism, and depression showed independent associations with the occurrence of falls. Among these, the strongest association was observed for depression.

The definition of falls in large surveys considers its occurrence in the 12 to 24 months preceding the interview, with a predominance of studies that cover the 12-month period. The prevalence found in ELSI-Brazil participants is within the international range and that observed in different Brazilian studies^2^ ^-^ ^10^. Regarding those who reported having fallen, 1.8% had a hip or femur fracture and, of these, about one third required surgery for prosthesis placement. These findings corroborate other studies that have identified this type of fracture as one of the most serious consequences of falls^9^ ^,^ ^10^. Falls have repercussions for the individual and their families, but also for the health system. In fact, the Brazilian public health system (SUS) spent more than one billion reais (i.e. about 250 million US dolars) on hospitalizations of older adults due to hip or femur fracture between 2002 and 2016^22^.

The association between older age and female gender with falls is consistent with most national and international studies on the subject^2^ ^,^ ^4^ ^-^ ^6^ ^,^ ^9^ ^,^ ^16^. Regarding gender, the greater longevity of women might explain this association, since a greater proportion of older women are exposed to different diseases^23^. In addition, older women usually present more unfavorable health and functional conditions, with greater fragility, obesity, and limitations in performing daily living activities, which may favor a greater probability of falls^2^ ^,^ ^4^. Regarding age , it is a consensus in the literature that, in older patients, the occurrence of falls is higher^2^ ^,^ ^4^ ^,^ ^5^, since the advancement of age increases the predisposition to loss of muscle mass and bone density, with consequent postural instability and changes in gait and balance, conditions associated with the occurrence of falls^4^.

Something new in this study refers to the analysis of the association between factors related to the urban environment and falls at the national level. In the last decade, the World Health Organization has launched the Global Age-Friendly Cities Project^24^, with recommendations for adapting the urban environment to the needs of older people. The objectives expressed in that document include safety for locomotion in the streets and buildings, accessibility, lack of barriers, and encouraging participation in civic and cultural activities and voluntary work. Our results are worrying. About half of the older adults in urban areas reported being afraid of falling due to defective sidewalks and fear of crossing streets, while a third considered their neighborhood very insecure due to violence. In this analysis, the fear of failing due to defective sidewalks and the fear of crossing the streets emerged as factors independently associated with the occurrence of falls. The rapid aging of the Brazilian population living in urban areas makes the adaptation of the urban environment to this new demographic context one of the priorities of public policies. The recent initiative of the Brazilian Ministry of Social Development, the Age-Friendly Strategy, developed with several partners^25^ to support the cities that are friendly to the aged, is in line with this concern.

The results of this analysis show that older adults with diabetes and with arthritis or rheumatism are respectively 17% and 20% more likely to have had one or more falls in the previous year. The association between falls and health conditions is in line with that shown in the literature^2^ ^,^ ^10^ ^,^ ^11^ ^,^ ^14^ ^,^ ^16^. This association can be explained by the complications of these diseases. In the case of diabetes, complications – such as decreased visual acuity and neuropathy – may lead older adults to decrease their activities and this restriction increases their risks of falling^2^. In addition, a recent study^26^ showed that sustained hyperglycemia is related to loss of muscle mass and strength, which may also justify the observed association. In relation to arthritis and rheumatism, the higher prevalence of falls can be explained by increased stiffness and joint pain, which lead to instability in walking and alterations in the balance^2^ ^,^ ^11^. As for depression, the health condition that showed the strongest association with falls, our results are in line with those observed in Ireland^15^ and in British men^4^, as well as in a meta-analysis that showed that older adults with depressive symptoms are 50 % more likely to fall^27^. In this analysis, the likelihood of falling was 53% higher among those who had had a medical diagnosis of depression.

Falls are health sentinel events in old age. The occurrence of a fall should signal to the health team the need for differentiated attention. Our results show the relevance of the health conditions for the occurrence of falls, emphasizing the importance of the health sector for the prevention and rehabilitation consequent to this aggravation. As a strategic action in this sector, we have pointed out the use of instruments that help primary care professionals and other care levels to identify both the occurrence of falls and the factors that may be related to them. In Brazil, the fourth edition of the Health Record of the Aged Person^28^ (Caderneta de Saúde da Pessoa Idosa), launched by the Ministry of Health in 2017, addresses the issue of falls, both from the identification of its occurrence and through illustrated guidelines for its prevention. The Record asks questions about the location of the fall and possible physical and emotional consequences. In a multidimensional assessment context, this information can be worked together with the environmental assessment and health conditions of the aged.. Other instruments that enable the identification and prevention of risk factors for falls can also be used as a health care strategy for the aged. In addition to identifying the occurrence of falls, it is important to implement preventive actions involving multidisciplinary teams^1^. Professionals working in different Primary Care settings (such as the *Núcleo Ampliado de Saúde da Família e Atenção Básica* [Nasf-AB] and the *Programa Academia da Saúde*) should plan preventive actions such as dance, Tai Chi Chuan, muscle strengthening exercises, balance and motor coordination exercises^29^, as well as other activities that may benefit the different dimensions of the health of older adults.

The main advantage of this study is its large population base and the inclusion of environmental aspects that had not yet been considered in fall studies. Among the limitations of the study, is obtaining information on falls by means of self-report, which, although usual in studies on the subject^2^ ^,^ ^4^ ^-^ ^7^ ^,^ ^10^, may underestimate its occurrence, since older adults may not remember the falls they considered to be of lesser gravity. Another limitation refers to the difficulty of establishing temporal relationships due to the cross-sectional nature of the study. For example, associations with the fear of falling and crossing streets may be consequences of previous falls, a phenomenon known as reverse causality. This, however, does not diminish the importance of our results, which can subsidize policies for improvements in the urban environment aimed at preventing further falls.

In summary, our results show a high prevalence of falls among older adults living in urban areas. Applying the prevalence of 25% to the absolute number of the population aged 60 years and over living in urban areas in Brazil (25 million)^30^, we estimate that approximately 6.2 million old people would have fallen in the last year. The findings also show that the factors associated with falls among older adults are multidimensional, pointing to the need for intra and intersectoral actions that consider the integrality of care for their prevention.

## Footnotes

a Fundação Oswaldo Cruz. Brazilian Longitudinal Study of Aging. Rio de Janeiro; c2015 [cited 2017 Nov 28]. Available from: http://elsi.cpqrr.fiocruz.br
